# Supplementary material for: Provisioning Australian Seed Carrot Agroecosystems with Non-Floral Habitat Provides Oviposition Sites for Crop-Pollinating Diptera
Source: Insects. 2023 May 4;14(5):439. doi: 10.3390/insects14050439 (PMC10231055; doi:10.3390/insects14050439)
Supplement: Supplementary file 1 [file insects-14-00439-s001.zip › insects-2371798-supplementary.pdf]

Supplementary information

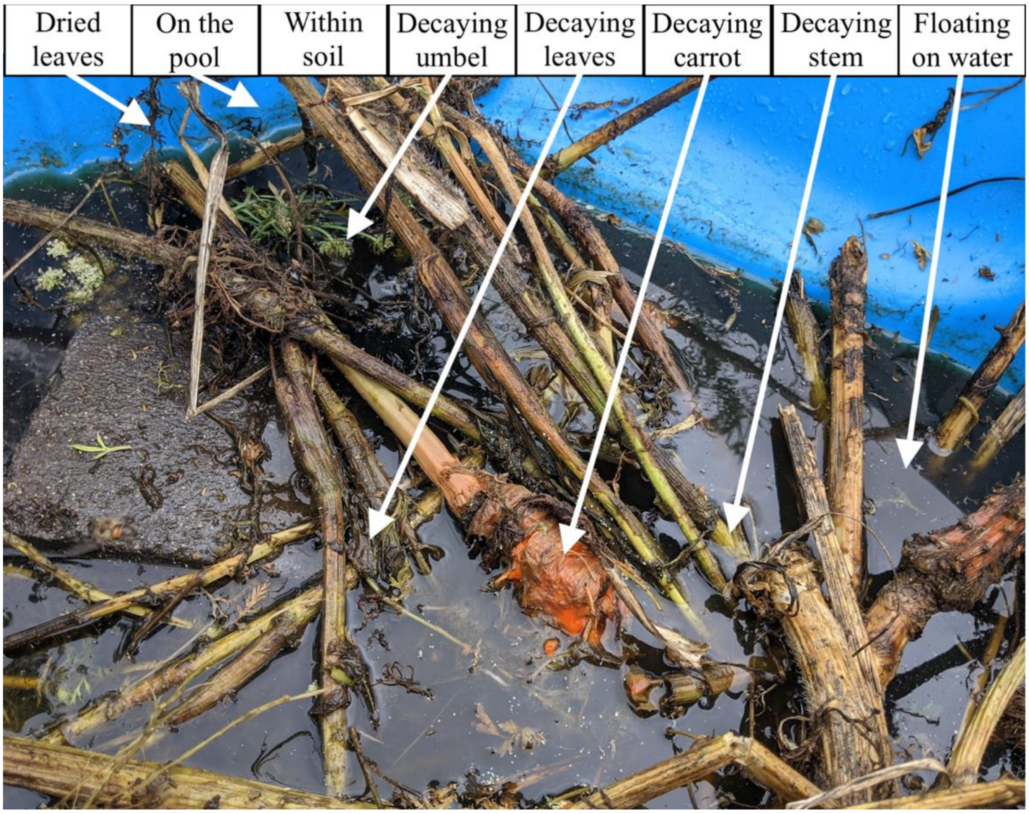

Figure S1: Locations of eggs within habitat pools. Locations within the deployed habitat pools where eggs were found. Arrows are used demonstrate where locations were within pools.

Table S1: Egg results. Generalized linear mixed effects model analyses of the number of eggs found within habitat pools differed based on habitat (carrot plants + water or soil + carrot plants + water), the number of days the pools were deployed, location (seven categories), or site.

Fixed effects	Random effects	Estimate	Std. error	df	z value	p value	Std. deviation	Variance
Habitat: carrot plants + water	n/a	2.277	4.046	6	0.563	0.5735	n/a	n/a
Habitat: soil + carrot plants + water	n/a	1.124	1.444	6	0.779	0.4360	n/a	n/a
Days	n/a	-0.0251	0.2345	4	-0.107	0.9146	n/a	n/a
Location: Decaying leaves	n/a	-1.713	1.275	7	-1.343	0.1791	n/a	n/a
Location: Decaying stems	n/a	2.487	1.167	7	2.130	0.0331 *	n/a	n/a
Location: Decaying umbel	n/a	-2.430	1.205	7	-2.016	0.04381 *	n/a	n/a
Location: Dried leaves	n/a	-5.310	1.442	7	-3.681	< 0.001 ***	n/a	n/a
Location: Floating on water	n/a	2.856	1.235	7	2.313	0.0207 *	n/a	n/a
Location: On the pool	n/a	-5.216	1.437	7	-3.630	< 0.001 ***	n/a	n/a
Location: Within soil	n/a	-3.011	1.486	7	-2.026	0.0428	n/a	n/a
n/a	Site	n/a	n/a	13	n/a	n/a	2.333	5.444

Table S2: Larval results. Generalized linear mixed effects model analyses of the number of larvae (living or dead) found within habitat pools differed based on habitat (carrot plants + water or soil + carrot plants + water), the number of days the pools were deployed, and site.

Fixed effects	Random effects	Estimate	Std. error	df	z value	p value	Std. deviation	Variance
Larvae: Dead	n/a	7.238	1.022	1	7.081	< 0.001 ***	n/a	n/a
Larvae: Living	n/a	2.603	0.4245	1	6.133	< 0.001 ***	n/a	n/a
Days	n/a	-0.4398	0.0664	4	-6.617	< 0.001 ***	n/a	n/a
Habitat: soil + carrot plants + water	n/a	-0.1866	0.3992	1	-0.468	0.64	n/a	n/a
n/a	Site	n/a	n/a	13	n/a	n/a	< 0.001	< 0.001

*** indicate significant differences (p < 0.001) between factors

Table S3: Larval instar pairwise comparisons. Tukey multiple pairwise comparison post-hoc test comparing the number of larval instars (first, second, and third) found within habitat pools based on the number of days the pools were left to decay at each site.

contrast	Estimate	Std. error	df	z value	p value
First instar – Second instar	0.65	0.498	1	1.305	0.3925
First instar – Third instar	1.18	0.510	1	-2.315	0.0537
Second instar – Third instar	0.53	0.516	1	1.026	0.5602
